# Postoperative Remote Acute Subarachnoid Hemorrhage as a Complication of Chronic Subdural Hematoma Evacuation With Burrhole: A Case Report and Literature Review

**DOI:** 10.1002/ccr3.71076

**Published:** 2025-10-01

**Authors:** Sadegh Bagherzadeh, Faramarz Roohollahi, Morteza Faghih Jouybari, Leila Bahari

**Affiliations:** ^1^ Department of Neurosurgery, Shariati Hospital Tehran University of Medical Sciences Tehran Iran; ^2^ Spine Center of Excellence Yas Hospital, Tehran University of Medical Sciences Tehran Iran; ^3^ Sports Medicine Research Center Neuroscience Institute, Tehran University of Medical Sciences Tehran Iran; ^4^ Department of Physical Medicine and Rehabilitation, School of Medicine Tehran University of Medical Sciences Tehran Iran

**Keywords:** burrhole, case report, review, subarachnoid, subdural

## Abstract

Chronic subdural hematoma (CSDH) is a common neurosurgical condition. Surgical evacuation, particularly burr hole trephination, is the preferred treatment method. However, postoperative complications such as hematoma recurrence and intracerebral hemorrhage may occur. Isolated subarachnoid hemorrhage (SAH) is rare but can occur after the evacuation of a CSDH. We presented the case of an 83‐year‐old male who underwent the double burr hole evacuation of the left CSDH and then had SAH in the immediate postoperative CT scan. The vascular study was negative, and the patient was treated medically for SAH, resulting in a full recovery. We also found four similar reports in the literature. This is a rare complication; no vascular lesions have been identified in recorded cases. Most cases recover completely, with one fatality reported. It's generally considered a benign condition. Our recommendations include strict perioperative blood pressure management, preventing rapid or excessive drainage, and reversing anticoagulants to avoid this complication.

## Graphical abstract

Post‐operative Axial Non‐enhanced brain CT scan of an 83‐year‐old male with chronic subdural hematoma, which was evacuated with burrhole craniotomy. The image shows a subarachnoid hemorrhage at the posterior fossa (left image), bilateral convexity (middle image). The basal cisterns are open despite the hemorrhage.

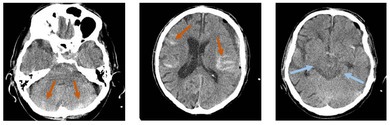

**Summary**

- Chronic subdural hematoma (CSDH) is commonly treated with burr hole evacuation, but rare complications like isolated subarachnoid hemorrhage (SAH) may occur.
- We present an 83‐year‐old male who developed SAH post‐CSDH evacuation, recovered fully with medical treatment, and reviewed similar cases.
- Recommendations include managing blood pressure, drainage control, and anticoagulant reversal.

## 1 Introduction

Chronic subdural hematoma (CSDH) is a common neurosurgical disease. Clinical presentations include headache, hemiparesis, drowsiness, and deterioration of consciousness. Nonetheless, sometimes CSDH can be asymptomatic [1]. CSDH also occurs bilaterally, and the clinical presentation is usually variable [2, 3]. Surgical evacuation is the most common treatment for symptomatic patients, typically resulting in significant neurological improvements. There are three techniques used for surgical evacuation: craniotomy, burr hole trephination, and twist‐drill trephination. Among these, burr hole trephination is the most favored due to its low recurrence rate and minimal patient morbidity [4, 5].

After surgery, the drain may be placed under the dura (subdural drain: SDD) or beneath the pericranium (subperiosteal drain: SPD). Theoretically, the SDD may carry a higher risk of brain parenchymal injury; however, a meta‐analysis found no significant differences in mortality or overall complication rates between SDD and SPD. The choice between SDD and SPD often depends on the surgeon's preference [6, 7].

The management of CSDH may initially appear straightforward and efficacious. However, postoperative complications such as hematoma recurrence, pneumocephalus, brain collapse, and intracerebral hemorrhage persist in certain patients. These complications are contingent upon the surgical approach employed, the patient's age, and any preexisting morbidities [8, 9]. Subarachnoid hemorrhage (SAH) refers to bleeding between the arachnoid and pia mater in the central nervous system (CNS). SAH is categorized as traumatic (due to an inciting event) or spontaneous (without an immediate identifiable cause). A further sub‐classification of SAH is aneurysmal SAH (aSAH) [10, 11].

Isolated SAH is an exceptionally rare finding after CSDH evacuation, with only a few cases reported in modern literature. Despite acute subdural hematoma and intracerebral hemorrhage being more frequently observed, we will present a rare case of SAH that occurred immediately after the evacuation of a CSDH. Additionally, we will review the relevant literature and discuss potential mechanisms for this complication.

## 2 Case History/Examination

### 2.1 History

An 83‐year‐old male patient presented to our emergency department with slurred speech and gait disturbance. He had a fall 2 weeks before, and his symptoms started 1 week before admission. He is currently taking Losartan 25 mg twice daily and Atorvastatin 40 mg daily for arterial hypertension. Additionally, he has been using Aspirin 80 mg every night for the past 15 years for the prophylaxis of ischemic heart disease.

### 2.2 Physical Examination

He was slightly obtunded with a Glasgow Coma Scale (GCS) score of 13 (Motor: 6, Eye: 3, Verbal: 4). His cranial nerves, sensory function, and deep tendon reflexes were normal, but he had a right‐sided weakness (Medical Research Council (MRC) Scale for Muscle Strength 4/5). He did not have urinary or fecal incontinence.

## 3 Methods (Differential Diagnosis, Investigations, and Treatment)

### 3.1 Diagnostic Evaluation

**FIGURE 1:**
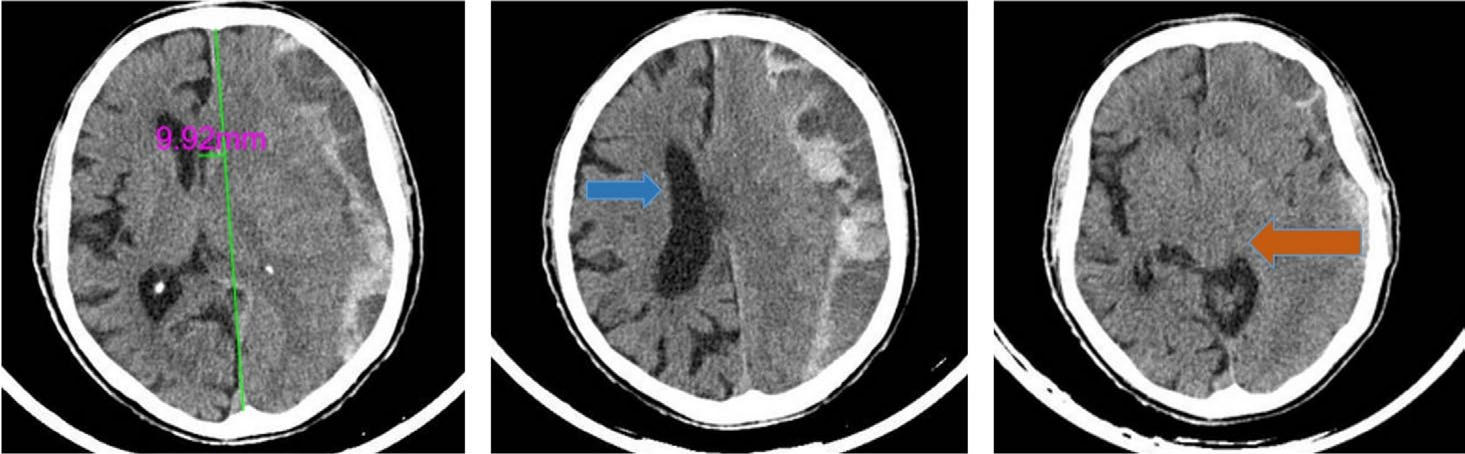
Preoperative Brain CT scan. Left image: The midline shift is about 10 mm. Middle image: The right lateral ventricle is visible (blue arrow), but due to hematoma, the left one is not seen, and the sulci on the left side are effaced. Right image: The red arrow shows that the left basal cisterns are compressed.

The brain CT scan revealed a mixed‐density collection on the left side with a thickness of 23 mm, indicating a trabeculated CSDH (Figure 1). This led to severe compression of the left lateral ventricle and almost complete effacement of the cortical sulci. There was a midline shift of 9.9 mm and compression of the left basal cisterns. His baseline coagulation tests, including a normal platelet count of 235,000 per microliter, activated Partial Thromboplastin Time (aPTT) of 32 s, and International Normalized Ratio (INR) of 1.34, were all within normal limits.

### 3.2 Therapeutic Intervention

**FIGURE 2:**
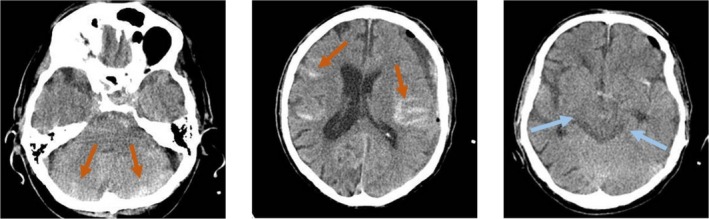
Postoperative Brain CT scan. Left image: Cerebellar subarachnoid hemorrhage. Middle image: Hemispheric subarachnoid hemorrhage. Right image: Basal cisterns are bilaterally symmetrically visible.

The patient was transferred to the Intensive Care Unit (ICU), made NPO (Nil Per Os), received six units of random donor platelets to reverse the effects of aspirin, and a cardiology consult was performed as a preoperative measure. After 8 h of admission (approximately 6 h of NPO), the patient was transferred to the operating theater and underwent surgery under general anesthesia. Two boreholes were placed on the superior temporal line, and about 130 cc of motor‐oil‐colored fluid emerged. Surgery was relatively uneventful and lasted about 1 h; at the end of the surgery, a subgaleal drain was placed. His postoperative CT scan showed diffuse supra and infra‐tentorial SAH (Figure 2). The patient was extubated 4 h after surgery in the ICU, but was still drowsy. We performed a CT angiogram, but it didn't reveal any vascular findings. The patient was treated medically with nimodipine 30 mg q6hr for 7 days and rehabilitation.

## 4 Conclusions and Results (Outcome and Follow‐Up)

**FIGURE 3:**
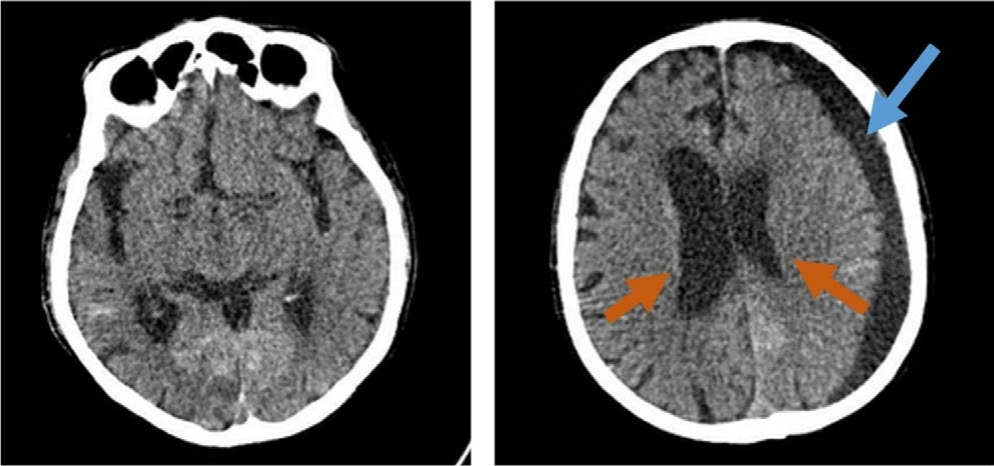
Second month postoperative Brain CT scan. Left: Basal cisterns are symmetric and open. Right: Both lateral ventricles are visible. The cerebral sulci are open, and there is a left‐side subdural hygroma.

The patient stayed in the ICU for seven days. His consciousness improved gradually, and he was able to eat and walk independently by day 10 postoperatively. He was discharged on the 12th postoperative day. Figure 3 depicts the brain CT scan of the patient from the second month of follow‐up.

## 5 Discussion

Table 1 presents the following data, summarizing five reported cases of Acute Isolated SAH after CSDH evacuation [2, 12, 13, 14]. Four patients fully recovered, while, unfortunately, one patient died. It is noteworthy that none of the patients had positive findings in vascular studies, suggesting the benign nature of this condition [15]. Three out of five patients had a Layered Gradated morphology on CT scan, a type that has a high chance of progression and recurrence [16]. All patients underwent burrhole craniotomy and had a drainage system; only two of five used anticoagulation drugs.

**TABLE 1:** This table shows the reported cases of subarachnoid hemorrhage after chronic subdural hematoma evacuation.

Author, year	Country	Age, sex	Anticogulant or antiplatelet	Presentation	CSDH side	CSDH type	Surgical treatment	Drainage (mL)	Vascular study	Outcome
Miyazaki et al. (2004) [12]	Japan	56, M	Warfarin	Sudden headache	Left	Layering Gradated	Single Burrhole	Not reported	Negative	Full recovery
Rusconi et al. (2015) [13]	Italy	62, M	None	Headache, Mental confusion	Right	Homogenous Isodense	Single Burrhole	650	Negative	Full recovery
Wang and Yu (2018) [14]	China	88, F	None	Progressive headache and dizziness	Right	Layering Gradated	Single Burrhole	420	Negative	Death
Corrivetti et al. (2021) [2]	Italy	64, M	None	Headache, Vomiting	Bilateral	Layering Gradated	Bilateral Single Burrhole	100	Negative	Full recovery
Index case	Iran	83, M	Aspirin	Right hemiparesis	Left	Trabculated	Double Burrhole	530	Negative	Full recovery

Abbreviation: CSDH, chronic subdural hematoma.

The possible causes of SAH occurring after burr‐hole craniotomy remain poorly understood. The proposed mechanisms of this complication include rapid overdrainage, hyperperfusion and cortical hyperemia, perioperative hypertension, and anticoagulant therapy. These factors will be reviewed in the following section.

### 5.1 Rapid Over‐Drainage

The rapid shift of the cerebral hemispheres caused by intraoperative evacuation or by the drainage system is considered the primary cause of this hemorrhagic complication. This theory is widely accepted and explains the occurrence of supratentorial SAH [17]. However, the Hyperperfusion theory may explain the occurrence of posterior fossa SAH in these patients. A rapid perioperative parenchymal shift could significantly worsen cerebral venous drainage. Sudden, copious drainage of hematoma fluid could lead to perioperative brain shift, which can tear the contralateral or other bridging veins, subsequently resulting in intracranial hemorrhage [18]. As part of our standard procedure to prevent rapid drainage in the operating theater, we place a cottonoid on the burr hole immediately after making the burr hole and opening the dura. This helps to slow down the drainage of subdural hematoma.

### 5.2 Hyperperfusion, Cortical Hyperemia

Brodersen and Gjerris [19] found that cerebral blood flow (CBF) decreases before surgery and then increases after surgery, as evidenced by intra‐arterial 133 Xenon CBF studies of CSDH patients. Koike's experiments demonstrated that rapid hemodynamic changes can cause leaky bleeding in intraparenchymal brain tissue. If CBF or cerebral autoregulation is impaired due to prolonged CSDH compression, a decrease in intracranial pressure from hematoma drainage can result in hyperperfusion [20]. Ogasawara et al. described this condition in the elderly, stating that long‐term brain compression by CSDH impairs vascular autoregulation, leading to a rapid decrease in intracranial pressure after hematoma drainage. Consequently, hemodynamic changes can cause hyperperfusion and cortical hyperemia, potentially leading to the rupture of a weak subarachnoid vessel [21].

### 5.3 Perioperative Hypertension and Coagulant Therapy

The theory was put forward by Miyazaki et al. [12]. However, as indicated in Table 1, the majority of patients did not utilize anticoagulants or antiplatelets. Conversely, when surgical intervention was pursued, these medications were discontinued. It appears that this theory has a limited impact on the development of SAH.

CSDH evacuation is a commonly performed procedure in neurological surgery, often carried out by junior residents (PGY‐1 and PGY‐2). While it is typically straightforward, there are instances of devastating complications. Although acute SAH is rare, it is essential to consider preventive measures to minimize the risk. Therefore, we recommend the following precautions:

– Complete reversal of anticoagulants and antiplatelets before surgery.
– Utilization of a cottonoid ball over the burr hole, as previously described.
– When using a drainage system, ensure that the zero is positioned at the midbrain level. The draining height should not be below this level. Additionally, precise tracking of the drainage amount is crucial; if necessary, the drainage should be clamped.

## 6 Conclusion

Our case highlights the rare occurrence of acute SAH following CSDH evacuation. While the exact cause remains unclear, we emphasize the importance of meticulous blood pressure management, controlled drainage techniques such as cottonoid use, and reversal of anticoagulants and antiplatelet agents.

## Author Contributions

**Sadegh Bagherzadeh:** conceptualization, investigation, visualization, writing – original draft, writing – review and editing. **Faramarz Roohollahi:** writing – review and editing. **Morteza Faghih Jouybari:** supervision, writing – review and editing. **Leila Bahari:** investigation, software.

## Ethics Statement

Our institution's ethical committee waived the requirement for ethical approval for this case report, as it was deemed part of standard patient care.

## Consent

The patient provided both verbal and informed written consent for the use of his clinical data and images in this case report; the manuscript and images do not disclose the patient's identity.

## Conflicts of Interest

The authors declare no conflicts of interest.

## Data Availability

The data that support the findings of this study are available on request from the corresponding author. The data are not publicly available due to privacy or ethical restrictions.
